# Peer Review of “Selection of the Optimal L-asparaginase II Against Acute Lymphoblastic Leukemia: An In Silico Approach”

**DOI:** 10.2196/33214

**Published:** 2021-09-08

*This is a peer-review report submitted for the paper “Selection of the Optimal L-asparaginase II Against Acute Lymphoblastic Leukemia: An In Silico Approach”.*

## Round 1 Review

### General Comments

Baral and coworkers [1] conducted a screening of L-Asparaginase II (asnB) for selection of asnB with increased asparagine depletion efficiency and decreased unwanted immune response for potentially improved efficacy of acute lymphocytic leukemia treatment in comparison to the commercially available asnBs. In their work, the asparagine hydrolyzation efficiency was assessed by the simulated asparagine binding energy, and the immunogenicity was assessed by the phylogenetic tree distance to the commercial asnB strains via molecular evolutionary genetics analysis. The three best asnBs out of 101 candidates were selected via the screening process. I found the overall work is somewhat of value. However, it can be improved by including some important specifications at each screening step.

### Specific Comments

#### Major Comments

1. The dissertation formatting is not the usual journal article type. Please normalize the introduction and the literature review section into one and make it concise and in a flow, such as (1) introduce the field of the work, its importance, and what has been done; (2) indicate a gap, a research question, or a challenge; and (3) clearly outline the research and its novelty.
2. Please specify the distance matrices used in phylogeny to produce a tree. Is it only sequence-based genetic distance or does it also include measured distance (ie, from immunological studies)? Sequence-based filtering, if lacking immunological factors, may bring in large inaccuracy in your case. If it is not included in the analysis, please suggest some literature references that show sequence-only–based filtering is sufficient to link to immunology. Otherwise, please thoroughly discuss the limitations.
3. At each screening step, please specify, among XX candidates, YY was selected, for ZZ reasons (eg, the distances is greater than AA from *E coli* K12; percent of residues in most favored regions is greater than BB%; the binding energy is greater than CC. This helps with clarifying and keeping track of the optimization.

#### Minor Comments

1. The tree plot is a bit hard to read. Please make it uniform and enlarge the font size in the same column and make it readable at 100% display. Please use squares rather than circles to highlight the candidates in the tree for better accuracy. Please explain what the numbers plotted on the tree branches are (bootstrap confidence levels?).
2. P15, line 299. Is it at the “top” or at the “bottom” of the tree? The current description does not match with the description in the figure legend.
3. Please include references in section 3.3 and in Table 1 for those identified active cites of asnBs from *E coli* and from other organisms.
4. In Table 2, do not use * as the multiplication sign (×). (Please also do not simply use the letter x for a correction.) In addition, please use a separate column for the references.

## Round 2 Review

### General Comments

All my comments have been addressed.

## References

[1] Baral A, Gorkhali R, Basnet B, Koirala S, Bhattarai HS (2021). Selection of the optimal L-asparaginase II against acute lymphoblastic leukemia: an in silico approach. JMIRx Med.

